# Comparison of Spectroscopy-Based Methods and Chemometrics to Confirm Classification of Specialty Coffees

**DOI:** 10.3390/foods11111655

**Published:** 2022-06-04

**Authors:** Verônica Belchior, Bruno G. Botelho, Adriana S. Franca

**Affiliations:** 1 The Coffee Sensorium Project, Av. Getulio Vargas, 159, Dores de Campos 36213-000, MG, Brazil; belchior.veronica@gmail.com; 2 PPGCA, Universidade Federal de Minas Gerais, Av. Antônio Carlos, 6627, Belo Horizonte 31270-901, MG, Brazil; 3 DQ, Universidade Federal de Minas Gerais, Av. Antônio Carlos, 6627, Belo Horizonte 31270-901, MG, Brazil; botelhobrunog@gmail.com; 4 DEMEC, Universidade Federal de Minas Gerais, Av. Antônio Carlos, 6627, Belo Horizonte 31270-901, MG, Brazil

**Keywords:** FTIR, NIRS, specialty coffee, PLS models

## Abstract

The Specialty Coffee Association (SCA) sensory analysis protocol is the methodology that is used to classify specialty coffees. However, because the sensory analysis is sensitive to the taster’s training, cognitive psychology, and physiology, among other parameters, the feasibility of instrumental approaches has been recently studied for complementing such analyses. Spectroscopic methods, mainly near infrared (NIR) and mid infrared (FTIR—Fourier Transform Infrared), have been extensively employed for food quality authentication. In view of the aforementioned, we compared NIR and FTIR to distinguish different qualities and sensory characteristics of specialty coffee samples in the present study. Twenty-eight green coffee beans samples were roasted (in duplicate), with roasting conditions following the SCA protocol for sensory analysis. FTIR and NIR were used to analyze the ground and roasted coffee samples, and the data then submitted to statistical analysis to build up PLS models in order to confirm the quality classifications. The PLS models provided good predictability and classification of the samples. The models were able to accurately predict the scores of specialty coffees. In addition, the NIR spectra provided relevant information on chemical bonds that define specialty coffee in association with sensory aspects, such as the cleanliness of the beverage.

## 1. Introduction

Brazil is the world’s largest coffee producer. The recent export data report that Brazil has shipped around 22.872 million bags (60 kg each) from July 2021 to January 2022. Specialty coffees accounted for 17.4% of total Brazilian exports, with an average price of USD 292.44 per bag, representing 23.4% of the total obtained with the shipments in January 2022 [1]. Specialty coffees, defined as high-quality products, are quite relevant for the coffee industry given the higher prices attained in comparison to commodity coffees. While a regular bag of regular green coffee costs approximately USD 200, specialty coffees can go up to USD 1000 per bag.

The quality of a cup of coffee begins in the field. Several factors including coffee species and variety, harvesting, post-harvesting conditions, blend elaboration, and roasting parameters, have a significant influence on the flavor and aroma of the drink. The delicate taste and aroma obtained from a cup of specialty coffee results from a complex combination of physical transformations and chemical reactions that start on the seed and end on the beverage preparation [2,3].

The most common way to evaluate the quality of a green coffee is by cup tasting [3,4]. Several industries, including perfume, coffee and tea, wine, beer, and tobacco, often employ trained personnel for sensory evaluation. In the specific case of coffee, such people are called “Q-graders” and trained to define the sensory profile of different samples. Then, according to the SCA (Specialty Coffee Association) protocol to evaluate coffee, they classify samples by giving different scores [5].

The SCA protocols are based on objective assessment methods, including the presence or absence of sweetness and defects, thus minimizing subjectivity compared to other methodologies. In addition, Q-graders are considered excellent and accurate in giving the scores related to quality, although some errors and inconsistencies regarding the description of a coffee are reported [6].

Furthermore, sensory analysis can lead to a few problems. Bias that comes from the preference and previous knowledge of a specific sample, as well as the influence of some external factors [6] can affect the analysis. Additionally, the Q-grader´s health during the cupping as well as modification on his (her) personal evaluation abilities over time can also affect the results. Such issues can be minimized by using alternative evaluation tools in order to make the coffee trading market more reliable [3,7]. Sensory analysis can also be viewed as a sensitive and time-consuming technique, given the need for well-trained personnel. Considering the economic relevance of specialty coffees in the world trade market, finding alternative tools to confirm coffee quality is of utmost importance.

Many studies have shown the potential of spectroscopic methods in food analysis, with near (NIR) and mid (FTIR) infrared among the most used methods [8]. The employment of such techniques for coffee analysis has been widely reported [9]. Applications include discrimination between coffee species and varieties [10], adulteration of roasted and ground coffee [11,12,13], and identification of low quality (defective) coffee beans [14,15,16]. Given that such low quality coffees have a significant effect on the sensory profile of the beverage, spectroscopic methods can also be used to detect differences in sensory parameters. In recent studies, our research group employed chemometrics to develop models for the classification and discrimination between espresso coffee beverages based on generic parameters (intensity and a few sensory aspects) informed by the manufacturers, and also based on sensory analysis performed by a trained panel [7,17]. It was also possible to develop models that classified coffees by cup quality parameters based on classification criteria that are specific to Brazil [4]. Some recent results from another group also showed the feasibility of mid-infrared and chemometrics to discriminate specialty coffees with different roasting profiles [18]. Our latest study showed that FTIR can be successfully used to discriminate specialty coffees classified by Q-graders [3], with models capable of predicting classification scores with high accuracy (validation coefficients above 0.97). Published studies confirm that both FTIR and NIR are promising techniques for coffee quality evaluation. However, in the case of NIR, only qualitative discrimination was performed with respect to coffee quality parameters given by Q-graders, without any attempt to provide an actual score-based classification. Furthermore, a comparison of spectroscopy-based techniques to evaluate specialty coffees has not yet been reported. Since both FTIR and NIR have been shown as reliable techniques for coffee quality definition, a comparison of these methods can indicate which method is more reliable. Although several studies have been described and tested with both techniques, there is still a need for further investigation, in order to improve the quality of predictive models to be applied for food quality evaluation [19].

Therefore, in this study, the potential of NIR was evaluated for establishing sensory characteristics of specialty coffees in terms of quantitative scores. Partial Least Squares (PLS) Regression was employed to build models in order to predict and establish a SCA-based sensory profile. NIR-based models were compared to FTIR ones that were developed in a previous study [3]. To the best of our knowledge, this is the first study in the literature that addresses such comparison for specialty coffee quality evaluation. Furthermore, this is the first work showing that NIR can provide quantitative quality scores.

## 2. Methodology

### 2.1. Roasting Tests and Sensory Evaluation

Arabica coffee samples submitted to dry (natural coffee) and wet (pulped natural coffee) processing were employed in the present study. Detailed information regarding sample provenance and quality scores (ranging from 81 to 91) is presented as Supplementary Materials (Table S1) and discussed in our previous study on FTIR analysis of specialty coffees [3]. A summarized description of sample preparation is presented as follows. The samples were roasted in accordance with the SCA protocol for coffee sensory analysis, using an IKAWA^®^ Sample Roaster Pro (London, UK). Individual samples consisted of 50 g of green coffee that were submitted to roasting at temperatures ranging from 170 °C to 227 °C. The roasting time was 4 min 34 s. Roasting tests were performed in duplicate. A total of 56 samples were obtained. These samples were ground using a Porlex Mini^®^ grinder (Porlex Grinders, Osaka, Japan) in order to obtain a fine and homogeneous grind (particle diameter below 0.150 mm). The samples were then analyzed by six professional Q-graders according to the SCA protocol. Twenty-four hours prior to cupping, the coffee samples were submitted to a light/medium roast (#55 to #65 Agtron color scale). Once the coffee was ground, fragrance and aroma was evaluated. Filtered water (93 °C) was added to the sample cup (five per sample), let to rest for 4 min, and then the beverage was tasted and evaluated according to the quality attributes established in the protocol [5]. Sample classification was based on global scores and aromatic descriptors established by the protocol. It is noteworthy that, given that the goal of this study to evaluate the performance of NIR in comparison to FTIR, the same set of samples was employed for both techniques.

### 2.2. ATR-FTIR and NIR Analysis

After roasting and grinding, the samples were analyzed on a Shimadzu IRAffinity-1 FTIR Spectrophotometer (Shimadzu, Japan) with a DLATGS (Deuterated Triglycine Sulfate Doped with L-Alanine) detector, using an ATR (Attenuated Total Reflectance) sampling device. The spectra were recorded in the wavenumber range of 3100–800 cm^−1^ and a total of 224 spectra were obtained (56 samples × 2 aliquots × 2 measurements). The NIR measurements were conducted in a Red-Wave-NIRX-SD Spectrophotometer (StellarNet Inc, USA) with 25µm diameter and RFX-3D reflectance base. Samples were transferred to a petri dish and placed over this base. The spectra were recorded within 900 to 2300 nm, 16 nm resolution, and 8 scans. Each roasted and ground coffee sample was analyzed in duplicate, totaling 112 spectra (56 samples × 2 measurements). The background spectra was based on the RS-50 reflectance disk. Both FTIR and NIR analyses were performed at room temperature (20 ± 0.5 °C) and all readings were based on roasted and ground (D < 0.15 mm) coffee samples.

### 2.3. Data Processing and Statistical Analysis

The software employed for statistical analyses were MATLAB^®^ software v7.9, 2009 (The MathWorks, Natick, MA, USA) and PLS Toolbox^®^ 6.7.1, 2012 (Eigenvector Technologies, Manson, WA, USA). The ATR-FTIR and NIR spectra were used as chemical descriptors in order to build the PLS models for prediction of the sensory analysis scores. The Kennard–Stone algorithm was used to divide the 224 FTIR spectra from FTIR into calibration (70%) and validation (30%) sets, and the same for the 112 NIR spectra. Orthogonal Signal Correction (OSC) and Mean Centering (MC) were applied for reducing the effect of noise, enhancement sample-to-sample differences, and removal of redundant information. The number of latent variables was defined according to the lowest RMSECV value obtained by Random Subset cross-validation. Model performance was measured by calculating the root mean square errors for both calibration (RMSEC) and validation (RMSEP) errors [3]. Selected models were the ones with the smallest RMSEC and RMSEP values [12].

## 3. Results

### 3.1. ATR-FTIR and NIR Analysis

**Figure 1:**
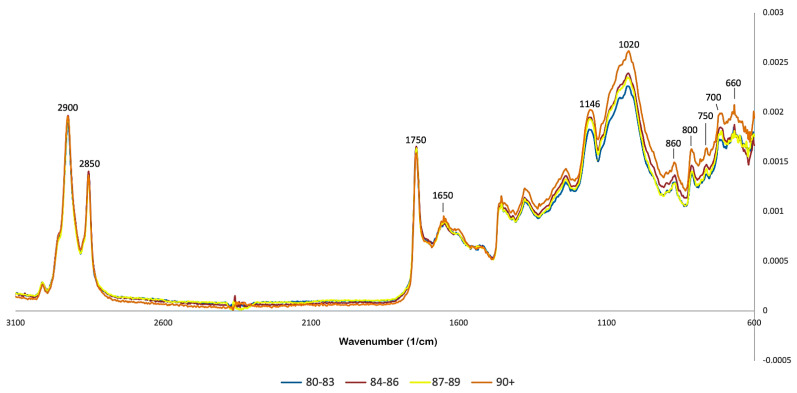
Average FTIR spectra obtained for roasted coffee (colors are related to sensory quality scores).

The spectra presented in Figure 1 represent the average FTIR spectra of four classes of samples grouped according to their score of sensory quality: 81–83; 84–86; 87–89; 90+. The two bands at the 2900–2850 cm^−1^ range are attributed to C-H vibrations of the bonds present in lipid and caffeine molecules [16]. The marked 1750 cm^−1^ and 1650 cm^−1^ regions are attributed to carbonyl (C=O) vibration and C=C bonds, attributed to carbohydrates and lipids, respectively [20].

The bands in the 1650–1600 cm^−1^ range have been previously reported in association with caffeine [4], and were employed in previous studies for quantitative analysis of this substance. The 1680–1630 cm^−1^ range has been found to be associated with vibrations in the carbonyl amide group [4] and also to the presence of trigonelline. The latter is usually decomposed into pyrroles and pyridines during roasting. Pyridines are some of the substances that are responsible for the characteristic aroma of roasted coffee [21].

A significant number of bands can be observed between 1500 and 900 cm^−1^. Carbohydrates have several absorption bands in the region between 1400 and 900 cm^−1^, also called “fingerprint region”, because it concentrates several relevant bands. The band at 1146 cm^−1^, has been linked to polysaccharides in previous studies, specifically to the C-O-C stretching of the glycosidic link in the cellulose molecule [4]. Bands in this have also been attributed to amino acids and proteins [22]. Nonetheless, an accurate chemical assignment of bands in this region is still a challenge because of highly coupled vibration modes of polysaccharide backbones [4]. The bands at the 1450–1150 cm^−1^ range have been reported in association with the presence of chlorogenic acids [16,22]. The band at 930 cm^−1^ has been previously reported in association with residues of 3,6-anhydro-galactopyranose [23] resulting from the thermal degradation of polysaccharides, such as galactomannan and arabinogalactan. The levels of chlorogenic acid and trigonelline as well as carbohydrate content will change significantly with roasting, so variations in the fingerprint region of the spectra are expected [14,24].

**Figure 2:**
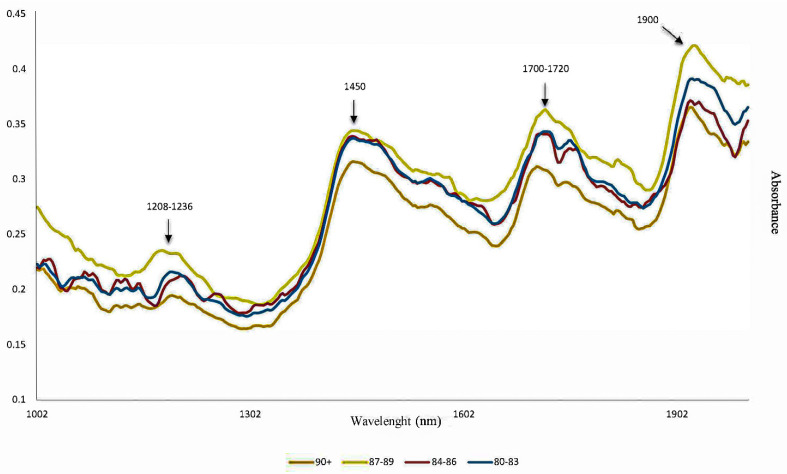
Full NIR spectra (1000–2000 nm) obtained for roasted coffee (colors are related to sensory quality scores.

Figure 2 shows the average NIR spectra of the samples, with different colors being associated with the sensory quality score. The most relevant bands present in the data are as follows: 1100–1250 nm (associated to CH, C-H2, and CH3 overtones from proteins, lipids, caffeine, and organic acids), and 1300–1490 nm (first overtones of RN-H of proteins, first overtones of OH of water and acids) [25]. The band in the 1900 nm region is associated with the combination of O-H angular stretching and deformation, related to the presence of water [26]. The region of 1208–1236 nm is the second bond overtone of C-H, C-H2, and C-H3, as well as the 1700–1720 nm region, which is related to the first overtone of the same carbon and hydrogen bonds, and C-H bonds linked to aromatic rings [26].

### 3.2. Partial Least Squares Regression (PLS)

**Figure 3:**
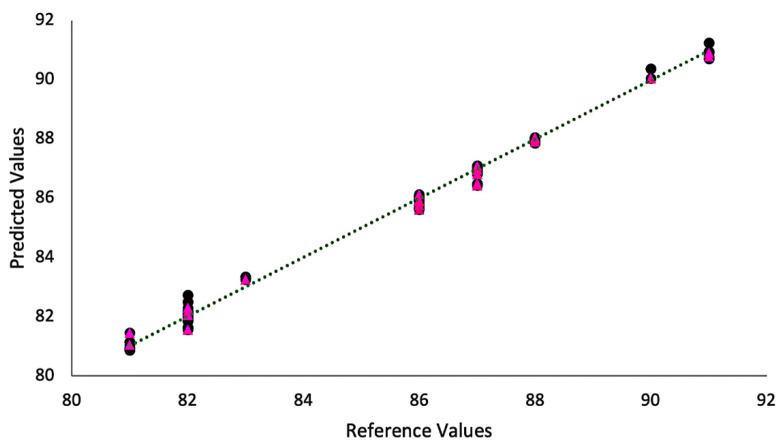
Experimental (black circles) vs. predicted values (pink triangles) obtained by the models based on FTIR spectra.

**Figure 4:**
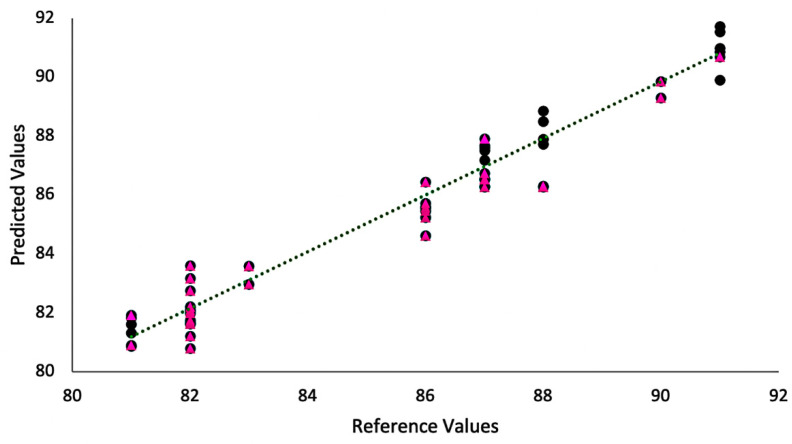
Experimental (black circles) vs. predicted values (pink triangles) obtained by the models based on NIR data.

Figure 3 and Figure 4 show the plots of measured vs. estimated values obtained for the models based on the spectra (estimated) in comparison to the quality scores provided by the Q-graders (measured). The models’ parameters are shown in Table 1. The FTIR model was built with two latent variables that were able to explain 99.71% and 81.2% of the accumulated variance in the spectra and sensory data, respectively. Both the values obtained for RMSEP and RMSEC were 0.23%, whereas calibration and validation coefficients were 0.99 and 0.97, respectively. In comparison, the NIR model used three latent variables that explained 90.4% of spectra data variance and 54.05% of the score (sensory) data. The RMSEC value was 0.50% and RMSEP value of 0.52%, and both the calibration (Rc) and validation (Rv) correlation coefficients were 0.98. Although both NIR and FTIR were able to provide good predictions, the FTIR results were slightly more accurate, given the smaller values for RMSEC and RMSEP and slightly higher values for calibration and validation in comparison to NIR. The potential of FTIR as a tool to classify specialty coffees was reported by Belchior et al. [3]. The comparison of both techniques highlights the efficiency of NIR as well. Nonetheless, despite its great potential in food analysis, the interpretation of the spectra in NIR analysis is challenging due to its broadband nature, which consists of overlapping overtone and combination bands [7,9]. The use of NIR-based models represents a new approach in comparing the chemical data with the SCA protocol for coffee classification. Although some studies have reported the use of NIR to evaluate coffees [9,25,27], the discrimination between high quality samples as well as the comparison with the SCA classification shown in this study confirms the potential of this method, providing the coffee industry with a good perspective for using different spectroscopy tools to evaluate coffee quality. Although previous studies [26,27] were able to show that NIR can be used to predict specific coffee sensory parameters (body, acidity, flavor, aftertaste, etc.), this is the first study that extends this application to quantifiable quality scores using an internationally accepted sensory protocol, thus confirming the potential of this method for coffee quality evaluation.

**Figure 5:**
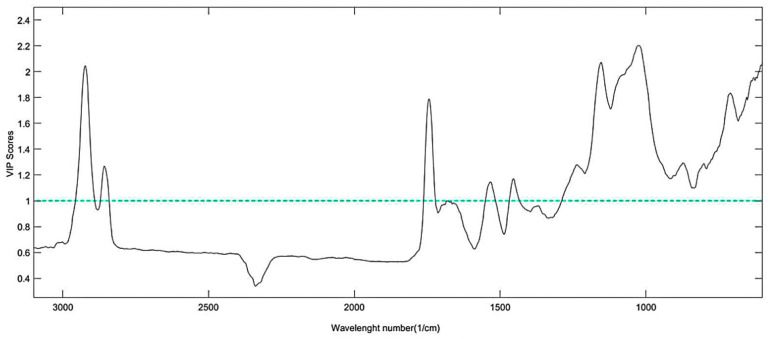
VIP Scores of the PLS models based on FTIR data.

**Figure 6:**
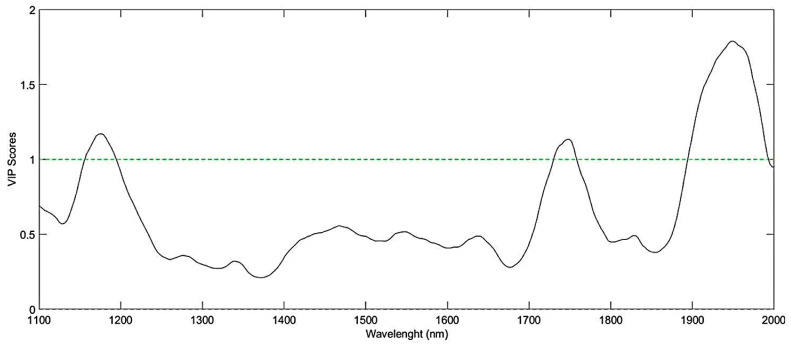
VIP Scores of the PLS models based on NIR data.

Figure 5 and Figure 6 show the Variable Importance of Projection (VIP) scores of the models. A VIP score is a measure of a variable’s importance in the PLS model and is calculated as the weighted sum of squares of the PLS weights, taking into account the amount of explained variance in each extracted latent variable (dimension). Thus, VIP scores above 1 are a typical rule for selecting relevant variables in a given model. An evaluation of the FTIR VIP scores (Figure 5) indicates that the entire spectrum affected the coffee classification in association with the SCA classification. The fact that bands in the whole wavenumber range were relevant in terms of sample classification indicates that many substances that are present in the coffee beverage have significant impact on the sensory profile. Besides coffee being a complex food matrix, several variables that affect the coffee processing chain processes (cultivation, harvesting, post-harvesting, storage, roasting, grinding, and extraction) will impact the final product and affect sensory variations that can be perceived by the Q-graders. Although roasting conditions are consistent and established in terms of the SCA protocol, there still can be variations in the roasting profile (environmental conditions, type of equipment, etc.) [28,29].

VIP scores obtained for the NIR model (Figure 6) show the bands 1176, 1749, and 1950 nm as the most relevant in predicting the coffee scores, being related to the second overtones of CH, C-H2, and CH3, first overtones of CH and C-H2, first overtones of OH, RCO2R, and CONH2, and second overtones of C=O [26]. These regions were assigned by Ribeiro et al. [26] as related to the sensory characteristics of the attributes: taste, cleanliness, and body of the beverage. The region comprised between 1156-1172 nm is attributed to caffeine, and 1738–1755 nm to the presence of lipids in the samples. The region between 1937–1959 nm are related to the ACG and water content of the samples. The beverage cleanliness, regarding the quality of body, is a relevant attribute evaluated in coffee and responsible for higher scores, reinforcing the feasibility of NIR data in adding more confidence to the results.

Schenker and Rothgeb [30] stated that the roasting process can be divided into three stages: drying, Maillard reactions, and development. Therefore, the sensory profile of the roasting coffee will be directly affected by roasting time because it is directly related to the specific phase of the roasting process. This will affect the final coffee composition in terms of several components and reactions, including chlorogenic acids and their derivatives, sugar caramelization, organic acids, volatiles, lipid migration, and melanoidin production; such composition will have a direct effect on the sensory profile [18,31,32]. Therefore, the possibility of validating the sensory analysis performed by Q-graders by using spectroscopic methods is quite relevant. The results obtained in this study are promising for the classification of specialty coffees and confirm the potential of both NIR and FTIR as fast and efficient alternatives for the task at hand. Furthermore, the results are of high interest to the coffee industry, bringing more confidence to the trading routine, given possible inconsistencies between classification of the same samples by sellers and buyers.

## 4. Conclusions

Spectroscopy-based methods, FTIR and NIR, were shown to be appropriate tools for confirming and predicting score classifications given by Q-graders to roasted specialty coffee samples. The results are promising from the chemometrics standpoint, with models presenting high values for calibration and validation correlation coefficients for both techniques, showing that NIR is also a good tool for predicting coffee quality. It is noteworthy that, even with all samples being of high quality, it was possible to discriminate the nuances in sensory profile. Although the analysis of the whole FTIR spectra of coffee seems to be slightly more efficient from a scientific point of view, NIR spectra also provided robust results related to relevant chemical parameters that define specialty coffee, such as the cleanliness of the beverage. NIR seems promising for routine analysis of specialty coffees, given its simplicity and the possibility of using portable equipment. Therefore, both techniques can be used to confirm and verify the coffee quality scores associated with the Q-graders assessment. As a result, the coffee industry would increase confidence in trading purposes, producing more consistent results. Nonetheless, further studies are needed in order to increase model robustness and applicability, given the intrinsic variations in coffee samples associated to geographical origin, edaphoclimatic conditions, cultivation, and processing techniques as well as variations in roasting parameters. The variability in roasting conditions and equipment in the case of commercially available roasted coffee samples, and the fact that the present methodology was not validated for such conditions, is noteworthy. One of the difficulties in using sensory analysis in the case of the coffee beverage is the need for strict control of roasting conditions in order to guarantee that the tasters will be able to perceive the flavors appropriately. The SCA protocol and the models herein used will be able to correctly classify specialty coffees prior to roasting, but are not suitable for samples that are already acquired as roasted coffees with varying degrees of roast.

## Figures and Tables

**Table 1:** Comparison of the PLS models for both FTIR and NIR techniques.

Model	FTIR	NIRS
Calibration set	149	74
Validation set	67	37
Latent variables	2	3
RMSEC	0.23	0.50
RMSEP	0.23	0.52
Rc	0.99	0.98
Rv	0.97	0.98

RMSEC = root mean square error of calibration; RMSEP = root mean square error of validation; Rc = calibration correlation coefficient; Rv = validation correlation coefficient.

## Data Availability

Data is contained within the article (or supplementary material).

## Supplementary Materials

The following supporting information can be downloaded at: https://www.mdpi.com/article/10.3390/foods11111655/s1, Table S1: Coffee sample provenance; sensory scores and description provided by the Q-graders.
