# Supplementary material for: Comparison of Spectroscopy-Based Methods and Chemometrics to Confirm Classification of Specialty Coffees
Source: Foods. 2022 Jun 4;11(11):1655. doi: 10.3390/foods11111655 (PMC9180846; doi:10.3390/foods11111655)
Supplement: Supplementary file 1 [file foods-11-01655-s001.zip › foods-1735112-SI.pdf]

Table S1 – Coffee sample provenance; sensory scores and description provided by the Q-graders

**Sample**	**Provenance (city)**	**Process**	**Coffee variety**	**Sensory Score (mean±SD)***	**Sensory Description**
A1	Serra do Salitre	Natural	Catuaí Vermelho	91.2±1.0	Floral, sweet notes, caramel, cocoa and red berries
A2	Araxá	Natural	Topázio MG1190	91.0±1.0	Jaboticaba** liquor, cognac, malt, dark chocolate, blackberry, grape, currant
A3	Coromandel	Natural	Bourbon Amarelo	90.4±1.5	Sweet caramel notes, tropical fruits, spices
A4	Patos de Minas	Pulped Natural	IAC 125 RN	88.7±1.0	Mainly sweet and caramel, milk chocolate notes
A5	Patrocínio	Pulped Natural	Acaiá do Cerrado	88.4±1.0	Milk chocolate, caramel, buttery
A6	Presidente Olegário	Pulped Natural	Bourbon Amarelo	88.2±1.0	Cocoa notes, red berries, sugary/caramel almonds
A7	Patos de Minas	Natural	Catuaí Vermelho	87.7±2.0	Milk chocolate, red berries
A8	Patrocínio	Natural	Bourbon Vermelho	87.6±1.6	Fruity, acid, caramel
A9	Campos Altos	Natural	Topázio MG1190	87.6±2.5	Milk chocolate, red berries

A10	Patrocínio	Natural	Bourbon Amarelo	87.2±1.5	Intense sweet aroma, milk chocolate
A11	Araxá	Natural	Catuaí Vermelho	87.0±1.5	Sweet caramel notes, almonds
A12	Araxá	Natural	Topázio	87.0±1.2	Fruity, passion fruit, mango, honey
A13	Campos Altos	Pulped Natural	Bourbon Amarelo	86.0±1.0	Milk chocolate, nuts, caramel
A14	Araxá	Natural	Bourbon Amarelo	86.0±1.5	Milk chocolate, caramel creamy mango, clean, sweet and delicate flavor
A15	Araxá	Natural	Catuaí Vermelho	86.0±1.5	Milk chocolate, caramel, blackberry, jaboticaba**
A16	Araxá	Natural	Catuaí Vermelho	86.0±1.2	Vanilla, chocolate, fruity, passion fruit notes
A17	Araxá	Natural	Topázio	83.0±0.8	Chocolate, hazelnut
A18	Campos Altos	Natural	Catuaí Amarelo	82.0±0.1	Nuts, caramel
A19	Carmo do Paranaíba	Natural	Catuaí Vermelho	82.0±0.3	Nuts, caramel
A20	Patrocínio	Natural	Mundo Novo	82.0±0.3	Nuts, caramel
A21	Patos de Minas	Natural	Catuaí Vermelho	82.0±0.3	Nuts, caramel

A22	Rio Paranaíba	Natural	Bourbon Amarelo	82.0±0.3	Nuts, caramel
A23	Coromandel	Natural	Catuaí Vermelho	82.0±0.4	Nuts, caramel
A24	Patrocínio	Natural	Catuaí Vermelho	82.0±0.5	Nuts, caramel
A25	Patrocínio	Natural	Catuaí Amarelo	81.0±0.2	Nuts, caramel
A26	Presidente Olegário	Natural	Catuaí Vermelho	81.0±0.2	Nuts, caramel, lacks complexity
A27	Rio Paranaíba	Natural	Catuaí Vermelho	81.0±0.3	Nuts, caramel
A28	Coromandel	Natural	Catuaí Vermelho	81.0±0.4	Nuts, caramel

*SD = standard deviation **Brazilian fruit – *“Plinia cauliflora”*
